# A Prospective, Randomized, Controlled Study to Evaluate the Effectiveness of a Fabric-Based Wireless Electroceutical Dressing Compared to Standard-of-Care Treatment Against Acute Trauma and Burn Wound Biofilm Infection

**DOI:** 10.1089/wound.2023.0007

**Published:** 2023-11-03

**Authors:** Rodney K. Chan, Kristo Nuutila, Shomita S. Mathew-Steiner, Victoria Diaz, Kristin Anselmo, Maria Batchinsky, Anders Carlsson, Nandini Ghosh, Chandan K. Sen, Sashwati Roy

**Affiliations:** ^1^ United States Army Institute of Surgical Research, Ft. Sam Houston, Texas, USA.; ^2^ Comprehensive Wound Center, Indiana University School of Medicine, Indianapolis, Indiana, USA.; ^3^ Metis Foundation, San Antonio, Texas, USA.

**Keywords:** burn, biofilm, wireless electroceutical dressing, clinical burn study, acute treatment

## Abstract

**Objective:**

Despite advances in the use of topical and parenteral antimicrobial therapy and the practice of early tangential burn wound excision to manage bacterial load, 60% of the mortality from burns is attributed to bacterial biofilm infection. A low electric field (∼1 V) generated by the novel FDA-cleared wireless electroceutical dressing (WED) was previously shown to significantly prevent and disrupt burn biofilm infection in preclinical studies. Based on this observation, the purpose of this clinical trial was to evaluate the efficacy of the WED dressing powered by a silver–zinc electrocouple in the prevention and disruption of biofilm infection.

**Approach:**

: A prospective, randomized, controlled, single-center clinical trial was performed to evaluate the efficacy of the WED compared with standard-of-care (SoC) dressing to treat biofilms. Burn wounds were randomized to receive either SoC or WED. Biopsies were collected on days 0 and 7 for histology, scanning electron microscopy (SEM) examination of biofilm, and for quantitative bacteriological analyses.

**Results:**

In total, 38 subjects were enrolled in the study. In 52% of the WED-treated wounds, little to no biofilm could be detected by SEM. WED significantly lowered or prevented increase of biofilm in all wounds compared with the pair-matched SoC-treated wounds.

**Innovation:**

WED is a simple, easy, and rapid method to protect the wound while also inhibiting infection. It is activated by a moist environment and the electrical field induces transient and micromolar amounts of superoxide anion radicals that will prevent bacterial growth.

**Conclusion:**

WED decreased biofilm infection better compared with SoC. The study was registered in clinicaltrials.gov as NCT04079998.

**Figure:**
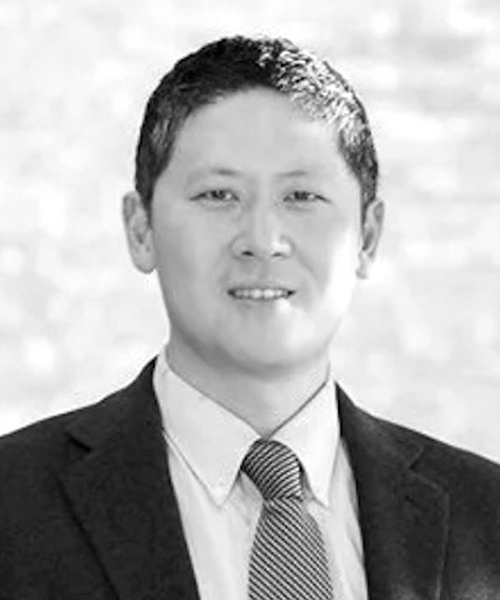
Rodney K. Chan, MD

**Figure:**
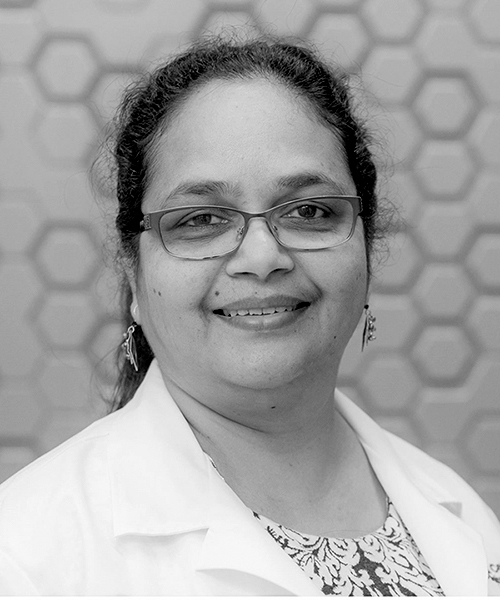
Sashwati Roy, PhD

## INTRODUCTION

Local management of infected burn wounds includes cleansing, debridement, topical antimicrobial agents (*e.g.*, silver sulfadiazine [SSD], combination antibiotics, chlorhexidine), and dressings. Of concern here is the notion that debridement may push the unseen biofilm deeper as was evident in our preclinical study where debridement was conducted by a burn surgeon.^1^ Our recent review provides more detail on biofilm management in wound care.^2^ Strategies to manage biofilm infection in wound care are typically based on: (a) adhesion inhibitors, (b) maturation (communication) inhibitors, and (c) biofilm disruptors. None of the physical, chemical, and biological agents/methods tested have been overwhelmingly superior that a single standard of care (SoC) exists. It is noteworthy that silver-based therapies are included among many of these possible SoC options.^3–8^

Ionic silver is bactericidal and a known inhibitor of microbes in the planktonic state.^3,9^ Therefore, studies that show favorable effects of silver on biofilm development are anticipated because bacteria are in planktonic state and therefore biofilms are inhibited. Some *in vitro* studies using silver on mature biofilms have identified that higher concentrations of silver are needed to disrupt mature biofilms.^8^ Although this may be suitable in *in vitro* conditions, the concern would be toxic effects of such high concentrations on host cells when used *in vivo*,^7^ impairing wound healing. In addition, other studies have shown that high silver concentrations are not needed, and that key is ensuring that silver can access the biofilm microbes. Silver-based dressings have been the *de facto* option since 1968 when 1% SSD was first used for burn wound infection care.^9^

The ease of use, availability, and familiarity associated with such dressings has led to their common application in burn wound infection management, although the evidence to support their beneficial effect is not overwhelming. In 2010, a Cochrane study of 26 randomized controlled trials (RCTs) and 2066 subjects performed between 1980 and 2008 (13 trials on burn patients) concluded that there was insufficient evidence to support the use of silver-based dressings and creams.^9^ A 2019 systematic review and meta-analysis of 11 RCTs that compared SSD with a comparative treatment showed that the comparators were statistically superior to SSD for mean time to burn wound healing.^5^ SSD could be detrimental to wound healing and increase hypertrophic scar formation.^10–13^

From the preclinical perspective, previous studies using a porcine burn biofilm model have shown that once biofilm is established, silver has limited benefit.^14,15^ At this time however, silver continues to be the preferred SoC treatment until novel therapies can be tested and accepted as superior alternatives.

The electrical basis of bacterial biology is an emergent discipline driven largely by the need for alternative methods of treating pathogenic strains.^2,16^ For a microbe living in environments (such as human body), this could provide survival advantages. Intrinsic electroactive pathways guide bacterial adhesion and cohesion, a key factor in the initial establishment of a biofilm.^17^ Once established, ion channel-based electrical signaling enables an advanced communication mechanism between bacteria of the same or different species.^18,19^ This guides growth dynamics and resource sharing in an environment where nutrient availability may be limited.

Furthermore, offense and defense mechanisms of microbial pathogens are dependent on electrical principles. For example, *P. aeruginosa* produces a redox-active, electrically charged molecule—pyocyanin (characteristic blue–green color in *Pseudomonas* infections)—that makes it more virulent (offense) to a human host.^20^ Extracellular polymeric substances (EPS) in a biofilm create a charged barrier (defense) that prevents the penetration of some antibiotics like aminoglycosides and glycopeptides.^21–23^ These processes are detailed in our 2020 review.^16^

In 1992, it was first reported that killing of biofilm bacteria by antibiotics can be dramatically enhanced by relatively weak (1.5 V/cm and 15 μA/cm^2^) electric fields.^24–28^ This “bio-electric effect” identified fundamental roles for electrical properties specific to bacteria. It was followed by several studies supporting the role for electrical interventions (*i.e.*, electroceuticals) as anti-biofilm and/or pro-healing.^24,25,29–43^ Most of these were performed under *in vitro* conditions. As we started to understand the fundamental role of electrical pathways in pathogenic microbial lifecycle, we identified a method of interrupting some of these pathways using an extrinsic weak electrical field generated in the form of an easy-to-apply dressing, applicable for wound care, that is, wireless electroceutical dressing (WED; commercially marketed as Procellera^®^ by Vomaris Innovations, Inc., Tempe, AZ).^29,44^

Our porcine studies where WED was tested on bacterial or fungal biofilm-infected burn wounds (10^6^ colony-forming units (CFUs); pathologic diagnosis for wound infection >10^5^ CFU/g of tissue^45^) showed that WED: (a) disrupted biofilm infection and (b) restored skin barrier function. The effect of WED on wound healing was consistent with the beneficial effects seen in keratinocyte and other cellular studies.^30,37,38,40,41,43,46,47^ The electrical output of this dressing is well within FDA permissible limits as outlined in FDA's electronic code of federal regulations 21 CFR 882.5890(c).^48^

Preclinical studies demonstrating the safety and efficacy of WED against bacterial biofilms^44^ lead to the study design of the current phase I, acute treatment trial. Since then, we have developed the next-generation dressing–Patterned Electroceutical Dressing (PED-10), with inherent scalability of electric field for dosing purposes, powered by a 6 V DC battery.

A pilot clinical study using PED-10 demonstrated that this dressing could be safely used in human chronic wounds as wound dressing.^49^ This prototype is currently in a randomized open-label clinical study (funded by the Department of Defense) to test the anti-biofilm efficacy in burn/trauma/surgery chronic wounds (NCT04794621). A search of PubMed^®^ identified various electrical stimulation methods used in clinical trials to study wound healing but not infection. Furthermore, none of these are comparative to WED because they are primarily based on electrical current rather than field.

Based on our published studies, we hypothesized that WED would reduce biofilm severity and infection load in clinically infected burn wounds. This first trial was designed to include a short-term treatment (1 week) with WED during which time SoC was withdrawn from the patient enrolled in a military burn center. This enabled testing of any potential adverse effects related to SoC withdrawal.

## INNOVATION

It is known that electric current inhibits bacterial growth. However, the current modalities of administering electric field technology as a therapy require a battery or an external power source. Thus, there has not been a practical way to administer electric current to mitigate biofilm infection in wound care. This study introduces a novel electroceutical dressing consisting of a silver–zinc electrocouple to generate a low electric field (∼1 V). The dressing becomes activated by a moist environment, inducing transient and micromolar amounts of superoxide anion radicals inhibiting bacterial growth.

## CLINICAL PROBLEM ADDRESSED

The American Burn Association (ABA) National Burn Repository shows that complications caused by infection represent 6 of 10 most common complications after burn injury.^1^ It is estimated that 42–65% of the mortality following thermal injuries is related directly to infections.^2–8^ Despite advances in antimicrobial therapy and the practice of debridement to manage bacterial load, bacterial infection remains a common problem in the management of burn injuries.^6^

## MATERIALS AND METHODS

### Study design

**Figure 1.**
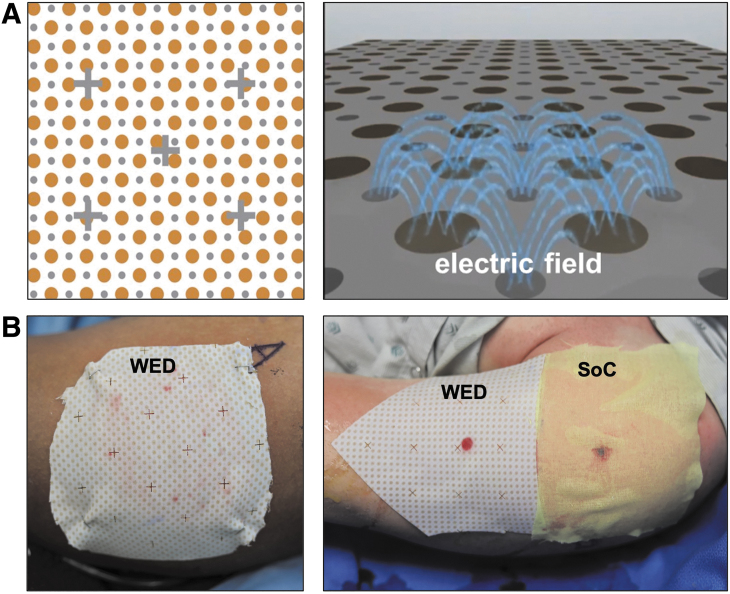
**(A)** WED consisting of a silver–zinc electrocouple to generate a low electric field (∼1 V) that induces transient and micromolar amounts of superoxide anion radicals (copyright permissions obtained; right panel image originally published in Banerjee *et al.*^32^). The dressing becomes activated by a moist environment. **(B)** The WED placed on burn wounds: Alone (*left*) and next to standard of care treatment (*right*). WED, wireless electroceutical dressing.

The study was designed as a single-center, prospective, randomized, controlled clinical trial to study the efficacy of WED (Fig. 1). The trial protocol was developed by the authors and was performed under an approved Institutional Review Board (IRB) protocol (C.2018.065) at the Brooke Army Medical Center (BAMC) and U.S. Army Institute of Surgical Research (USAISR) Burn Center (San Antonio, TX). All procedures were performed in accordance with the relevant guidelines and regulations of these centers.

### Participants

Inpatients admitted to the BAMC and USAISR Burn Center in San Antonio with dermal burn/traumatic wounds (Table 1 and Supplementary Table S1) were recruited based on the inclusion/exclusion criteria described in Table 2. All participants provided written informed consent.

**Table 1.** Demographic Characteristics of Study Participants

Characteristic	Number
Total number of patients who completed the study	34
Age, years	
Mean	45
Range	23–84
Sex	
Female	9
Male	29
Type of burn	
Chemical	1
Degloving	1
Electrical	5
Thermal/flash/steam/scald/flame/grease	29
Explosion/blast	1
Total body surface area (%)	
Mean	9
Range	2.5–28

**Table 2.** Study Inclusion and Exclusion Criteria

Inclusion criteria
Patients 18–65 years of age
Patient is willing and able to provide informed consent
Patient has acute wound(s) caused by trauma or burns meeting the following parameters:
Single wound ≥300 cm^2^ in size in one contiguous area or two separate wound sites ≥150 cm^2^
Exposure of deep dermis, subcutaneous tissue, muscle, fascia, tendon or bone
Exclusion criteria
Pregnancy
Prisoner
Current systemic steroid use
Active malignancy or immunosuppressive therapy
Know allergy or sensitivity to silver or zinc
Patient's proposed study wound site has any of the following conditions:
Location is on the hands, face or feet
Exposure of visceral organs
Exposure of hardware or prosthetic exposure

### Sample size

**Figure 2.**
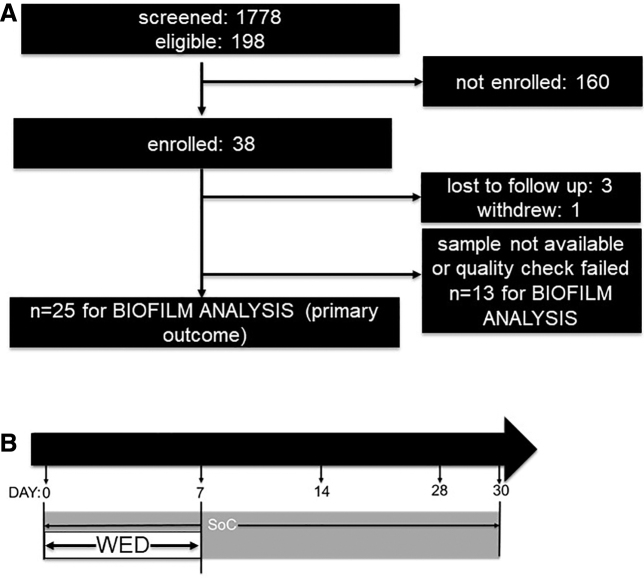
Study enrollment, randomization, and follow-up. **(A)** Of the 38 subjects with burns that completed the short-term study, 25 had complete data sets for further analysis. **(B)** Treatment design: For the first 7 days following burn, following randomization, patients received either SoC or WED at the site of burn. After this period of treatment, WED was discontinued and SoC alone was applied until completion of the study at day 30. SoC, standard of care.

Patients (*n* = 1778) receiving treatment at BAMC or the USAISR Burn Center were screened for inclusion in the study by review of the electronic medical record by research staff under a partial waiver of HIPAA authorization applicable to the recruitment phase of this study. No protected health information was collected for purposes of recruitment. Of these, 198 subjects met eligibility criteria of which a total of 38 subjects were enrolled in the study (Fig. 2). Complete data sets for the primary outcome (*i.e.*, biofilm analysis) were obtained from 25 subjects (for *n* = 9 subjects, the tissue samples did not meet quality check [QC] criteria for further analysis). For the secondary outcome (*i.e.*, infection load) analysis, 21 subject samples were included (for *n* = 6 subjects, tissue QC failure resulted in samples not being considered for statistical analysis).

### Randomization

On day 0, the injured area that was either one contiguous wound ≥300 cm^2^ or two separate but similar wounds with size ≥150 cm^2^ each was divided into two areas and marked “A” and “B” for the study treatment sites. Subsequently, the sites were randomized by opening a sealed envelope indicating the site that should receive either the SoC treatment or the WED dressing.

### Study procedures

Following randomization, procedures were performed as indicated in Table 3. Baseline assessments including clinical signs of infection and wound photography were completed. In addition, 3 mm punch biopsies of the treatment areas were obtained on days 0 and 7. After the baseline assessments, the dressings were applied according to the randomization. The dressings were changed on days 4 and 7 and the wounds were photographed, assessed for clinical signs of infection, and biopsied. Since this was the first study testing WED in clinical subjects, the study was designed to include acute treatment with WED for a 7-day period out of an abundance of caution. During this period, subjective assessment for safety and side effects was performed by research nurses. The wounds were visually assessed for obvious skin reddening and irritation. Subjects were verbally asked by the research nurses if they had any unusual feeling (tingling, pain *etc.*) in the area treated with WED.

**Table 3.** Study Design and Research Procedures

Research procedures	Intervention period			
	Visit 1	Visit 2	Visit 3	Visit 4
	Screening/pretreatment	Day 0	Day 4 ± 1 Day	Day 7 ± 2 Days
Assessment of eligibility	X			
Informed consent	X			
Medical history	X			
Physical exam	X			
Vital signs	X	X	X	X
Dressing application/change		X	X	X
Wound and infection visual assessment	X	X	X	X
Wound photography		X	X	X
Wound biopsy		X		X
Assess for adverse events		X	X	X

WED treatment was stopped at day 7. Subjects continued to participate in research visits at days 14 and 30 and wound closure (visual and TEWL) measurements and scar assessments was performed. These data are shown in Supplementary Data Content.

TEWL, transepidermal water loss; WED, wireless electroceutical dressing.

### Interventions

SoC included, but was not limited to, silver nylon, SSD ointment, bacitracin, xeroform, 5% sulfamylon solution, and Manuka honey as determined by the attending physician/provider. Regardless of randomization, the wounds still underwent SoC medical therapies including debridement, negative pressure wound therapy (NPWT), skin grafting, or other adjuncts as deemed necessary by the treating medical team. The test intervention was the WED (Procellera; Vomaris Innovations, Inc.),^29,30,44,50–52^ which is a polyester dressing with a geometrically printed matrix of elemental silver and zinc nanoparticles that generate a weak electric field upon contact with a conductive medium (*e.g.*, wound fluid or hydrogel) (Fig. 1). In this study, WED treatment was performed for 7 days (Fig. 2B) and therefore the analytical outcomes assess the acute/short-term impact of WED on burn wound biofilm compared with SoC alone.

### Blinding

For purposes of rigor, blinding of samples collected from the subjects was performed by the researchers at BAMC/USAISR. Each sample was given a number together with A or B (*e.g.*, sample collected from patient 1 was labeled 1A or 1B). The samples were shipped to the analysis team at Indiana University (IU) and processed for histological, biofilm, and microbiological analyses blinded. The blind was only removed after the entire study, including analysis, was completed.

### Scanning electron microscopy and quantitation

The samples were collected in glutaraldehyde fixation buffer, dehydrated with graded ethanol, and treated with hexamethyldisilazane (HMDS; Ted Pella, Inc., Redding, CA) and left overnight for drying.^14,44,53^ Before scanning, samples were mounted and coated with gold. Imaging of the samples was carried out using an FEI™ NOVA nanoSEM scanning electron microscope (FEI, Hillsboro, OR) equipped with a field-emission gun electron source.

**Figure 3.**
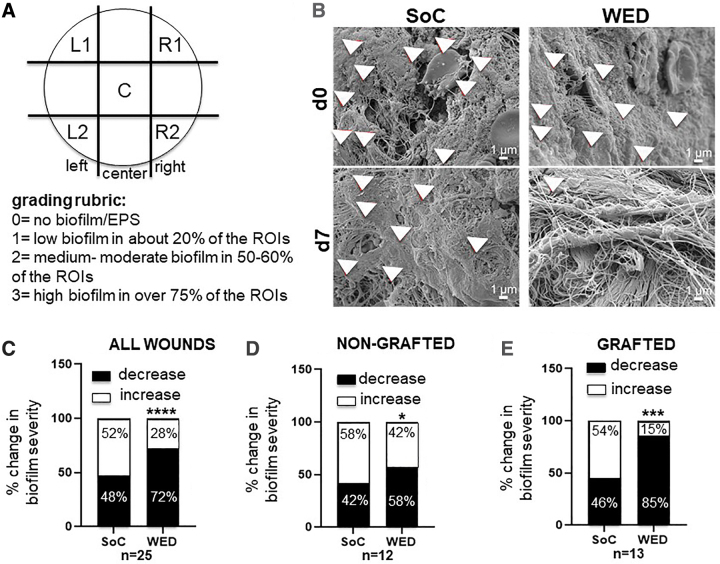
WED significantly decreased biofilm severity in all burn patients. **(A)** Biofilm grading rubric. Each sample was divided into five ROIs (LI, L2, C, R1, R2) as shown. Multiple images (*n* = 3–4) were taken from each area to minimize bias. Grading was performed following a 0–3 grade rubric where 0 = no biofilm or EPS and 3 = abundant biofilm and EPS in >75% of the sample. **(B)** Representative SEM images from day 0 and 7 of SoC or WED-treated burn patients. *Red arrowheads* point to biofilm EPS matrix containing bacteria. Blinded biofilm grading was performed on SEM images obtained from **(C)** all wounds (*n* = 25), **(D)** nongrafted wounds (*n* = 12), and **(E)** grafted wounds (*n* = 13). Change in grade between days 0 and 7 are represented as percentages in the graphs. WED-treated burn patients had significantly lower biofilm grade compared with SoC alone. Contingency analysis using Fisher's exact test was performed. *p < 0.05, ***p < 0.0001, ****p < 0.00001. EPS, extracellular polymeric substances; ROI, region of interest; SEM, scanning electron microscopy.

For scanning electron microscopy (SEM) quantitation, the blinded samples were imaged following a standard protocol (Fig. 3A). This protocol included division of the sample into five regions of interest (ROI) (top left [L1] and right [R1], center [C], bottom left [L2] and right [R2]). Multiple images were taken from each ROI to minimize bias. SEM biofilm grading was performed using a 0–3 scale rubric where 0 = no biofilm or EPS matrix visible in any of the ROIs, 1 = low biofilm with few bacteria and associated EPS matrix in ∼20% of the ROIs, 2 = medium–moderate level bacteria associated with bacterial EPS in 50–60% of the ROIs imaged, and 3 = high biofilm where abundant bacteria associated with bacterial EPS is visible in >75% of the ROIs imaged.

### Clinical microbiology and speciation

Biopsies collected at days 0 and 7 were sent to the IU Clinical Pathology Laboratory for standard bacterial culture-based analysis on tryptic soy agar. Bacterial load was evaluated using a standard semiquantitative quadrant streak method where 0 represented no colony/growth, 1 = growth on quadrant 1 (+), 2 = growth on quadrants 1 and 2 (++), 3 = growth on quadrants 1–3 (+++), and 4 = growth on all four quadrants (++++). Bacterial speciation was performed by matrix-assisted laser desorption ionization–time-of-flight mass spectrometry (MALDI-TOF) using an MALDI biotyper^®^ (Bruker Daltonik GmbH, Bremen, Germany). *Note: This method is agnostic to ‘unculturable’ microorganisms that will not grow under these standard conditions and will be lost to identification.*

### Outcomes

Primary outcome was to show that significantly higher percentage of WED-treated samples showed decreased biofilm severity compared with SoC treatment measured at day 7 using SEM (gold standard for biofilm analysis) imaging and quantification of wound biopsies. Secondary outcomes were to show that a significantly higher percentage of WED-treated samples showed decreased overall infection compared with SoC treatment measured at day 7 using standard clinical microbiological analyses. In addition, impact on visual and functional wound closure and wound quality (scar outcomes) was assessed secondarily with the following caveat: the direct impact of acute WED treatment (only for 7 days) on these long-term outcomes cannot be objectively evaluated.

### Sample size estimation

This trial was designed to determine the preliminary efficacy of the WED at preventing and treating biofilm infection. We assumed based on prior research^14,44^ that the biofilm infection rate for SoC dressings is 60% and thus the response to treatment is 40%. We assumed that the WED dressing will be successful if it achieves a 65% response and thus a 35% biofilm infection rate. Using a single-stage phase II trial design, we would require ∼40 evaluable patients. We estimated that the response rate is <40% if we observe 11 or more responses with error rate of 0.088, or we estimated that the response rate is >65% if we observe 10 responses or less with error rate of 0.185.

### Secondary assessments

Wound Closure: (a) SilhouetteStar measurement: The wounds were photographed with the SilhouetteStar™ camera (Aranz Medical Ltd.). The detailed images generated were used for wound closure assessment using the Silhouette Connect™ software. (b) Transepidermal water loss (TEWL): TEWL is a noninvasive measurement of the quantity of water that passes from inside a body and can be used to assess restoration of the skin barrier function. TEWL was measured from the wound and normal skin (baseline) using DermaLab TEWL Probe (cyberDERM, Inc., Broomall, PA) as published by us.^1,42^ TEWL was expressed in g/m^2^/h. Scar assessment: Quality of healing was assessed on days 14 and 30 using the Patient/Observer Scar Assessment Scale (POSAS) as well as the Vancouver Scar Scale (VSS).

Both observer and patient portions of the POSAS were completed at each assessment, with each variable measured as compared with normal skin. The same two independent observers completed the observer portion of the POSAS at each assessment to maintain consistency and to improve the reliability of the results. A score from 1 to 10 was assigned to each variable, with a score of 1 reflecting normal skin and 10 the worst scar imaginable. In addition, using the VSS the quality of healing was rated according to four parameters: vascularity (0–3), pigmentation (0–2), pliability (0–5), and height (0–3). Each parameter ranked subscales were summed to obtain a total score ranging from 0 (normal skin) to 13 (worst scar imaginable).

### Statistical analyses

All statistical analyses were performed using GraphPad Prism (GraphPad Software, Inc., La Jolla, CA). The frequencies of the categorical variables were compared using two-tailed Fisher's exact test or *χ*^2^-test as appropriate. A value of *p* < 0.05 was considered significant.

## Results

### Trial participants

A total of 38 subjects with burns were recruited for the study between February 28, 2019 and December 3, 2020. Four subjects did not complete the day 7 time point and one withdrew. Of the remaining 33 subjects, samples collected from 25 subjects (12 nongrafted and 13 grafted) had complete set of data (for days 0 and 7) for statistical analysis. Thirteen samples were considered incomplete because one or both biopsies collected did not meet minimum quality requirements for biofilm and/or microbiological analysis. The flowchart of the study design is given in Fig. 2. Demographics of the subjects are listed in Table 1.

### WED significantly decreased biofilm incidence and severity

WED-treated burn wounds had lower incidence of biofilm infection as imaged by SEM and quantitated following a grading rubric (Fig. 3A, B). In more than half (52%) of the 25 burn wounds treated with WED, little to no biofilm could be detected by SEM. Compared with this, only 24% of the pair-matched SoC-treated wounds showed lower biofilm incidence. Furthermore, WED significantly (*p* < 0.05) lowered or prevented increase of biofilm in all wounds compared with the pair-matched SoC-treated wounds (Fig. 3C–E).

### WED significantly decreased bacterial infection and incidence of select species

**Figure 4.**
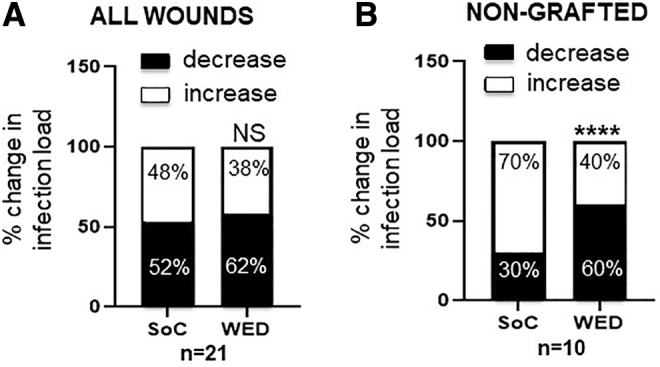
WED significantly decreased bacterial load in nongrafted burn patients. Blinded semiquantitative scoring of bacterial load was performed on **(A)** all wounds (*n* = 21) and **(B)** nongrafted wounds (*n* = 10). Change in score between days 0 and 7 are represented as percentages in the graphs and showed that nongrafted, WED-treated burn patients had significantly lower bacterial load compared with SoC alone. Contingency analysis using Fisher's exact test was performed. ****p < 0.0001; NS = not significant.

**Figure 5.**
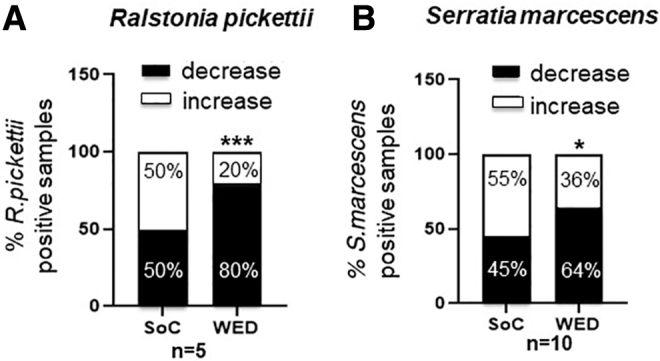
WED significantly decreased the incidence of specific opportunistic bacterial pathogenic strains in burn patients. Blinded semiquantitative scoring of incidence of **(A)** *Ralstonia pickettii* (*n* = 5) and **(B)** *Serratia marcescens* (*n* = 11) was performed. Percentage of samples containing the bacterial strains between days 0 and 7 is represented in the graphs. WED-treated burn patients had significantly fewer *R. picketti* (***p < 0.0001) or *S. marcescens* (*p < 0.05) compared with SoC alone. Contingency analysis using Fisher's exact test was performed.

In nongrafted burn wounds treated with WED, overall incidence of infection was significantly lowered or stalled compared with pair-matched SoC controls (Fig. 4). Of interest, two bacterial species that are well-known opportunistic pathogens—*Ralstonia pickettii* and *Serratia marcescens*—were particularly found to be responsive to WED compared with SoC treatment (Fig. 5).

### WED treatment was safe on human burn wounds

No study dressing-related adverse events were noted during the treatment period. The research nurses visually evaluated the wounds during dressing change (day 4) and end of WED application period (day 7) and did not observe any increased reddening or irritation of skin to indicate adverse reaction to WED. Verbal responses from subjects related to unusual occurrences such as tingling, pain, and discomfort in areas treated with WED indicated that none of these were present.

### Secondary data: WED does not adversely affect burn wound closure and quality

**Figure 6.**
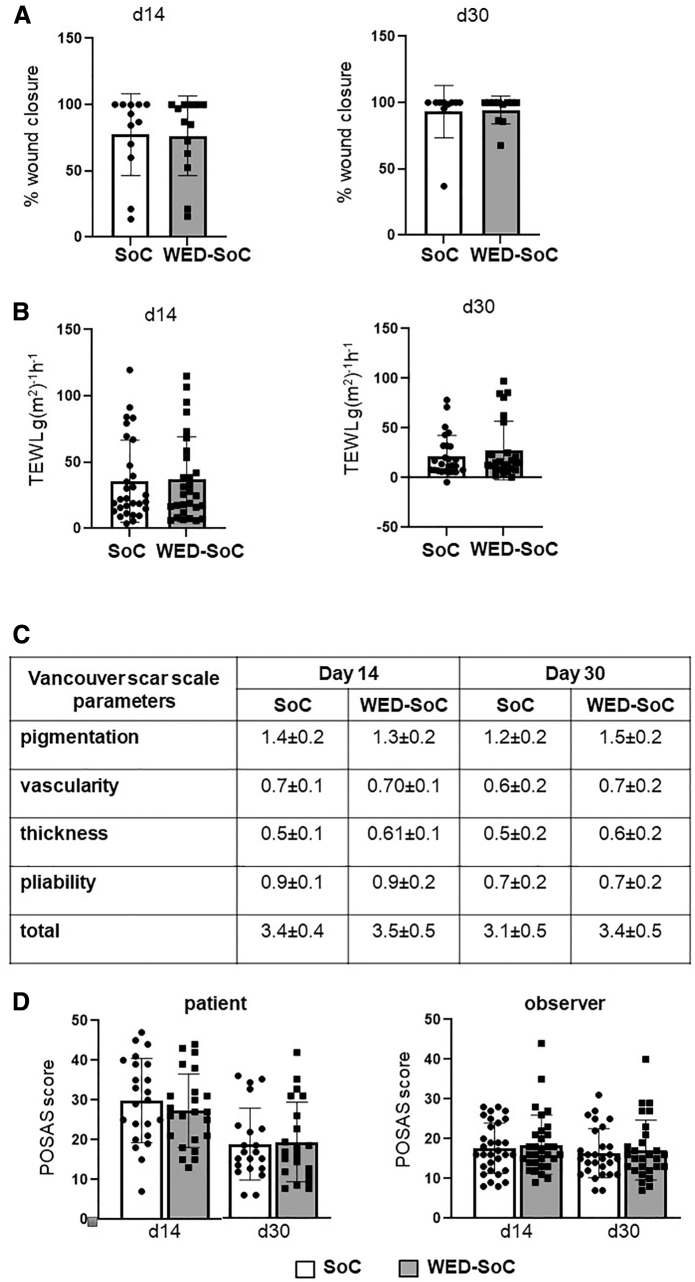
Acute treatment with WED does not adversely affect burn wound closure or quality. **(A)** Quantitation of wound closure at days 14 and 30 using digital planimetry. **(B)** Quantitation of TEWL at days 14 and 30. No significant differences were observed. WED-SoC = WED treatment. **(C)** Vancouver Scar Scale Measurements, **(D)** POSAS. POSAS, Patient Observer Scar Assessment Scale; TEWL, transepidermal water loss.

Acute, short-term (7 days) treatment with WED did not significantly impact long-term outcomes such as wound visual (planimetry) or functional (TEWL) closure (Fig. 6 shows quantitation of SoC and WED-treated wound at days 14 and 30 post-treatment onset). Similarly, no difference in quality of wound, that is, scar formation was noted (Fig. 6 shows quantitation of VSS and POSAS outcomes).

## DISCUSSION

**Figure 7.**
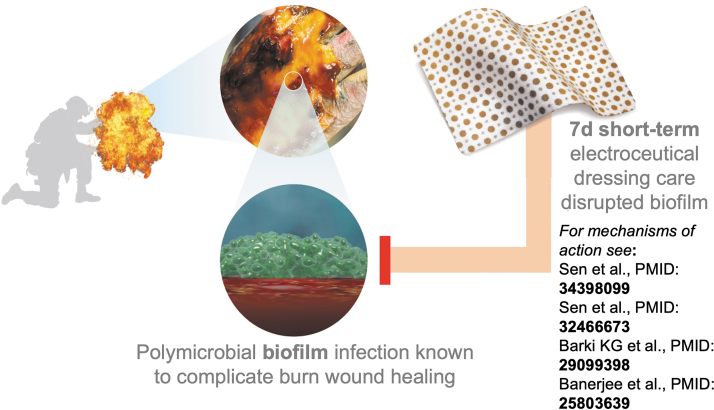
Summary graphic. In this study, patients with burn wounds admitted to the Brooke Army Medical Center (San Antonio TX), were subject to short-term (7 days) treatment with a WED. WED was more efficient in inhibiting biofilm infection compared with standard of care. Proposed mechanisms of action are described in prior publications from this group. The images were purchased and used per standard agreement policy from iSTOCK.com.

In this prospective, randomized, controlled clinical trial, we have shown that early, short-term treatment with WED significantly decreased biofilm severity in burn wounds (Fig. 7). These outcomes are consistent with published studies using WED^44^ in a preclinical porcine model showing significant decrease in bacterial biofilm. At present WED is the only dressing of its kind that uses electric principles for anti-biofilm applications. WED is easy to apply, can be combined with other procedures such as grafting and NPWT,^51^ and has no reported side effects (none identified in preclinical,^44^ published,^51^ and current clinical study). In a study presented at the Symposium on Advanced Wound Care, it was shown that WED-treated wounds showed a 52% average reduction in therapy cost per patient compared with advanced SoC. WED is already FDA cleared (510(k)#-K180533) for prescription and over-the-counter use and are commercially available.

The product is also registered and being commercialized in several countries outside the United States. *Indications for Prescription Use:* intended for the management of wounds to provide a moist wound environment and is indicated for partial and full-thickness wounds such as pressure ulcers, venous ulcers, diabetic ulcers, first- and second-degree burns, surgical incisions, donor and recipient graft sites, and so on. *Indications for Over-the-Counter Use:* intended for the management of wounds to provide a moist wound environment and is indicated for superficial wounds such as minor cuts, scrapes, irritations, abrasions, blisters, and so on. *Contraindications:* WED is not to be used on individuals with sensitivity or allergy to silver, zinc, or other dressing components. Therapeutically, WED would be applied alone or together with other dressings, NPWT, grafts and left on for up to 7 days at a time based on quantity of exudate from the wounds.

Calibrated approaches are needed to test alternatives to SoC such that room is made for product innovation while not adversely affecting patient safety. An important consideration when designing this study was whether SoC treatment should be withheld while subjects were enrolled.^54,55^ The answer to this will be study specific. Primary limitations of this study are the short window of treatment with WED and the relatively small subject population in the final analysis. The impact of the 7-day WED treatment on long-term outcomes such as wound closure (visual and functional) and scar formation is hard to objectively assess in this context. In this study, subjects had acute burn wounds that required frequent examination and possible debridement and grafting. Results of this study provide objective rationale for such withholding of SoC in a future patient-based study testing WED.

Secondary data (Fig. 6) show that the 7-day treatment with WED followed by continued SoC did not adversely impact the wound healing and scar outcomes. Prior clinical studies have addressed the impact of WED (also called microcurrent dressing or bioelectric dressing) in promoting faster wound closure in the context of various wounds.^56–58^ Immediate next steps should include studies with longer duration of WED treatment in the context of clinical burns to address the direct impact with respect to wound closure and scar outcomes.

There are a few studies that have used Procellera™ in various clinical study settings but not in the context of biofilm infection. Most of these have been in the context of wound healing, scarring, and patient outcomes from patients undergoing skin grafting^57^ and Army Ranger trainees with blisters.^59^ Two studies studied impact on rate of healing and showed that Procellera-treated wounds generally heal faster^58^ requiring fewer dressing changes and lowering cost of treatment.^51^

This study investigates the effect of WED on biofilm and that is not a current indication under FDA clearance. This work corroborates the anti-biofilm efficacy of WED in burn wounds and supports the safety of use in the context of human subjects. A larger clinical trial with longer duration of treatment investigating the impact on wound healing outcomes in relation to biofilm infection management is warranted.

**KEY FINDINGS**

- The WED is a simple, easy, and rapid method to protect the wound, while also inhibiting infection.
- WED-treated wounds had significantly less biofilm compared with the SoC-treated wounds.
- This therapy is not specific to any particular organism, unlike antibiotics.

## Supplementary Material

Supplemental data

## ABOUT THE AUTHORS

**Rodney Chan, MD** is the chief of plastic and reconstructive surgery at BAMC. **Kristo Nuutila, MSc, PhD** and **Anders Carlsson, PhD** are Principal Research Scientists at the Department of Combat Wound Care at the USAISR. **Victoria Diaz, RN** and **Kristin Anselmo, RN** are research nurses at the Metis Foundation. **Maria Batchinsky, BS** is a research trainee at the USAISR. **Shomita S. Mathew-Steiner, MSc, PhD** is Associate Director, Scientific Operations at IU School of Medicine (SoM). **Chandan K. Sen, PhD, FNAI** is Distinguished Professor and J. Stanley Battersby Chair of Surgery at IU SoM. **Sashwati Roy, PhD** is Professor of Surgery at IU SoM.

## Abbreviations and Acronyms

ABA: American Burn Association
BAMC: Brooke Army Medical Center
EPS: extracellular polymeric substances
HMDS: hexamethyldisilazane
NPWT: negative pressure wound therapy
POSAS: patient observer scar assessment scale
QC: quality check
RCT: randomized controlled trial
ROI: region of interest
SoC: standard of care
SSD: silver sulfadiazine
TEWL: transepidermal water loss
USAISR: U.S. Army Institute of Surgical Research
VSS: Vancouver Scar Scale
WED: wireless electroceutical dressing
