# Supplementary figures and images for: A Prospective, Randomized, Controlled Study to Evaluate the Effectiveness of a Fabric-Based Wireless Electroceutical Dressing Compared to Standard-of-Care Treatment Against Acute Trauma and Burn Wound Biofilm Infection
Source: Adv Wound Care (New Rochelle). 2023 Nov 3;13(1):1–13. doi: 10.1089/wound.2023.0007 (PMC10654645; doi:10.1089/wound.2023.0007)

## Slide 1
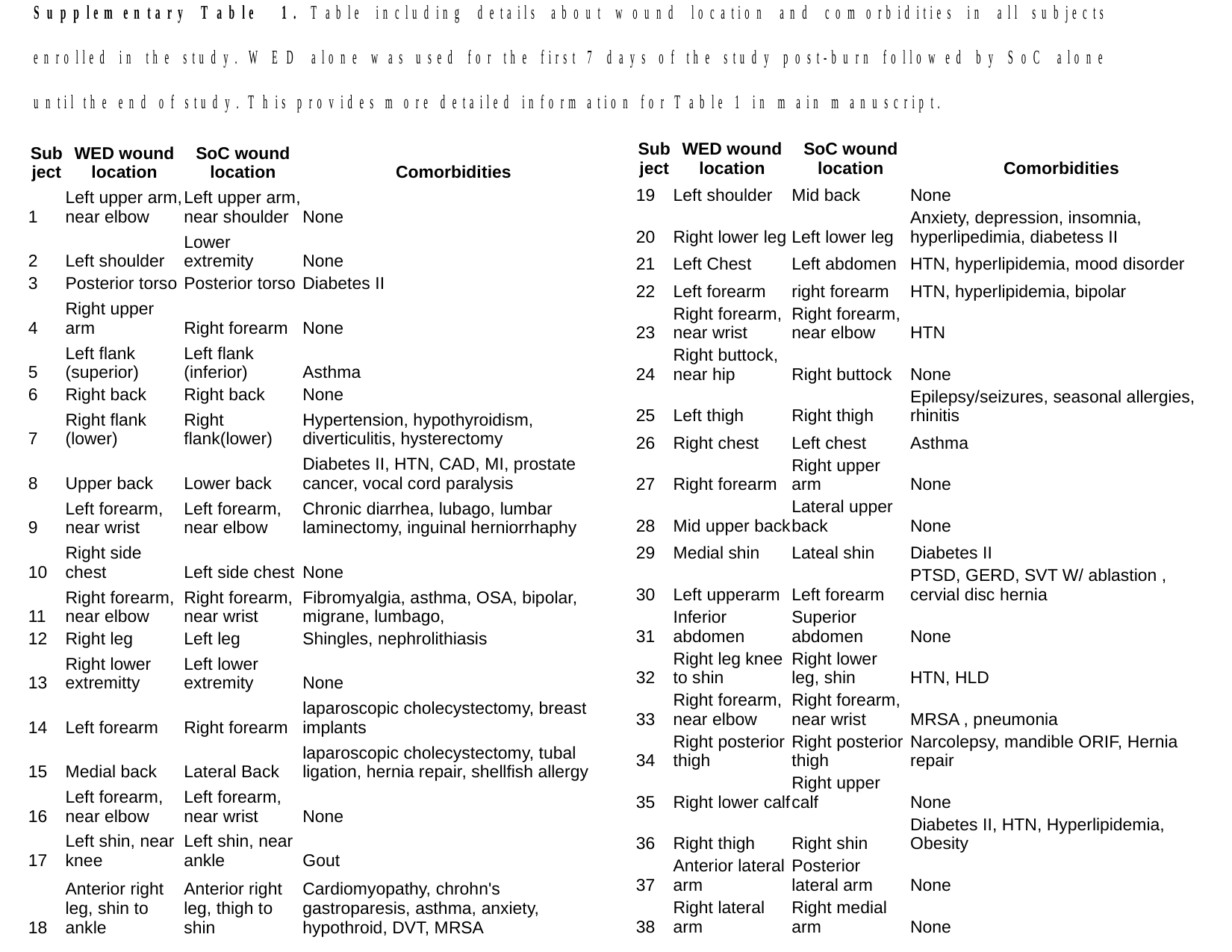

Supplement: Supplemental data [file Suppl_TableS1.pptx]
